# Managers’ and Administrators’ Perspectives on Digital Technology Use in Regional Long-Term Care Homes During the COVID-19 Pandemic

**DOI:** 10.1177/23337214221146665

**Published:** 2023-01-10

**Authors:** Asif Khowaja, Nawal Syed, Kaitlyn Michener, Kristin Mechelse, Henriette Koning

**Affiliations:** 1 Brock University, St. Catharines, ON, Canada; 2 Niagara Region Seniors Services, St. Catharines, ON, Canada

**Keywords:** digital technology, older adults, COVID-19, pandemic restrictions

## Abstract

In this paper, we explore managers’ and administrators’ perspectives on digital technology use for residents during province-wide lockdowns (June–August 2021) during the COVID-19 pandemic in seven regional long-term care homes (LTC) in Niagara, Canada. Fifteen semi-structured interviews were conducted with participants representing operational, financial, and recreational departments where we discussed their needs and factors influencing the use of digital technology during the phases of increased restrictions on visitors and social isolation. Our findings indicate extensive use of cellular devices including smartphones, however additional iPads were needed to meet the ever-rising demand for virtual connections. Almost all participants revealed supportive leadership, redeployed staff, and community donations as main facilitators for technology use. Barriers related to managing varying elderly cognitive capacities and technical issues affected technology use. Based on our findings, we conclude that financial commitment and community support are integral for future-proofing LTC homes with technological innovations.

## Introduction

The Coronavirus (COVID-19) pandemic continues to pose an unprecedented threat to Canadians’ health, social, and economic well-being (Public Health Agency of Canada, 2020). From a global perspective, as of August 22, 2020, Canada ranked #79 out of 210 countries concerning total cases per million inhabitants and #26 for total deaths per million (Ritchie et al., 2020). The disproportionately high ranking based on fatality rates was largely attributed to outbreaks in long-term care (LTC) homes in Canada (Tam, 2020). While residents and staff were both affected, compromising 15% of overall cases, residents alone accounted for 80% of all COVID-19 related deaths associated with LTC (Tam, 2020). Additionally, other factors such as overcrowding in some LTC homes, old infrastructure with poor ventilation, chronic understaffing, and shared healthcare workers between multiple homes were identified as contributors to the spread of COVID-19 transmission (Tam, 2020). In response, on March 13th, 2020, an infection prevention policy was announced including visitor restrictions which later transitioned into partial and complete facility lockdowns to minimize the risk posed by asymptomatic individuals spreading the virus (Liu et al., 2020). As a result of increased restrictions on visitors and volunteers of LTC homes, the impacts of social isolation on elderly residents also increased (Chu et al., 2020). Studies from various elderly populations reported an increase in anxiety, and depression, especially those facing extended lockdowns. However, these effects were magnified in the elderly population mainly due to stricter lockdowns, the higher threat of illness, and the loss of social support (Ahmed et al., 2020; Martins Van Jaarsveld, 2020). Other studies have shown that depression in the elderly is linked to subsequent cognitive decline, and risk of Alzheimer’s disease (Geerlings et al., 2000). Therefore, while the lockdowns posed an immediate threat of social isolation to elderly residents, the long-term effects could be devastating.

In order to address issues related to social isolation, several LTC homes embarked on the use of digital devices to engage and interact with residents remotely by means of teleconferencing apps such as Skype, Facetime, or Zoom (Chu et al., 2020). Consequently, some older adults increased their typical use of technologies during the pandemic due to the need to adapt and connect with others (Sin et al., 2021). Yet, other older adults experienced barriers to increased technology adoption (Martins Van Jaarsveld, 2020; Sin et al., 2021). For example, one study found that a transition to using online resources required some older adults in Saudi Arabia to increase their dependence on physical human help to manage daily activities, which exacerbated the risk of infection (Alharbi et al., 2021). Also, other studies suggested that some older adults felt forced to increase their use of technology out of a need to connect leading to less autonomy (Nurain, Caldeira, et al., 2021). It was also noted that older adults faced tensions with this feeling of reduced autonomy and increased safety concerns during the pandemic (Nurain, Caldeira, et al., 2021). Further, it has been observed that the introduction of telehealth initiatives (i.e., the act of providing healthcare digitally, and remotely) has had fewer positive effects on the elderly compared to the general population. A recent study showed that about 40% of elderly individuals were unprepared to use telehealth resources, predominantly due to a lack of skills to effectively use the technology (Lam et al., 2020).

Although research findings on technology-related needs and uptake in the LTC population are emerging, current research is narrow in scope, focused only on resident-level implications, and directed by specific, researcher questions (Nurain, Caldeira, et al., 2021; Sin et al., 2021). There is a need for more research to take a broader view of the organizational decision-making for technology adoption, operational challenges, and enabling factors, that are most important to promoting the access and utilization of digital technologies in LTC homes during the COVID-19 pandemic and other crisis situations. A comprehensive understanding of what and how technology solutions were rolled out during lockdowns, from a management perspective, is warranted for effective planning, implementation, and scaling up of future technology-focused innovations to support older adults in LTC homes. In this study, we explore administrators’ and managers’ perspectives on needs, enabling, and impeding factors associated with digital technology use during the COVID-19 pandemic-related lockdowns in LTC homes.

## Methods

A qualitative exploratory study design was applied (Dermody et al., 2021), and the data were prospectively collected through semi-structured interviews with 15 LTC home managers and administrators from June to August 2021. There are eight publicly funded LTC homes across the Niagara Region in Southern Ontario, Canada. These LTC homes are generally categorized by bed capacity (i.e., three small-size homes included ≤80 residents, three mid-size homes included 81–140 residents, and two large-size homes included ≥141 residents).

Study participants were recruited using purposive sampling based on their expertise and knowledge related to administrative/operational/financial decision-making in LTC homes. The purposive sampling strategy ensured that the perspectives were information-rich and relevant to this study topic (Etikan et al., 2016). To recruit, we sent study invitation emails to managers and administrators in all eight LTC homes and two follow-up reminders were sent out one week apart after the initial invitation. One large-size LTC home did not respond to our invitation due to an active outbreak of COVID-19 cases and strict mandatory provincial regulations/lockdowns at the time of data collection. The inclusion criteria were that participants be 18 years of age or older, hold an administrative/managerial position (i.e., Chief Financial Officer, Program Manager, Senior Financial Analyst or Administrator), and had worked for at least 1 year preceding the time of data collection. Given the visiting restrictions in LTC homes, the interviews were conducted virtually using teleconferencing applications such as Zoom and/or Microsoft Teams. We excluded participants that did not use/check emails to respond to our invitation or had no access to a computer/smartphone to participate in the interview. This study obtained institutional ethics approval (REB file # 20-363) and written informed consent was obtained from each study participant prior to the interviews.

**Figure 1.**
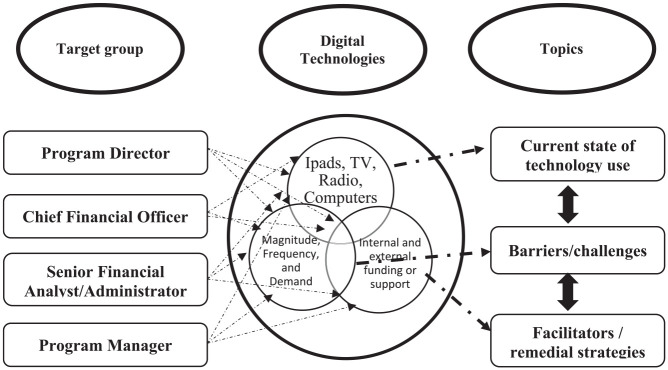
Perspectives on digital technology-use in long-term care homes.

The semi-structured interview guide was developed based on key areas identified in the Conference Board of Canada (CBC, 2011) Report focusing on elements of an effective innovation strategy for LTC in Ontario. The interview guide consisted of five to six leading questions (along with two to three probes per leading question) and focused on the following topics: the current use as well as needs/demands of digital technologies, facilitators/enabling factors, and barriers/impeding factors influencing the uptake of digital technologies in the selected LTC homes (Figure 1). We anticipated one/two participants from each LTC home (i.e., a proxy representation of all LTC homes across the region). The desired number of interviews, however, was determined based on the data saturation (i.e., we continued interviews until no new information was revealed in the interviews). Out of the fifteen interviews, five interviews were conducted from small-sized homes, three interviews were conducted from mid-sized homes, and three interviews were conducted from large-sized homes. Additionally, four interviews were conducted with senior executives overseeing all LTC homes in the region. Fourteen participants identified as females, and one identified as male. Participants work experience ranged from 1 to 15+ years (AVG ~4 years) in the current position.

The interviews were conducted by a graduate student research assistant (NS) and the duration of each virtual interview lasted about 45 **minutes.** The audio-visual recordings were undertaken for each interview and the transcripts were auto-generated using the teleconferencing application. The written notes were taken during each interview, and these were later used to spot-check transcripts for consistency and spelling errors. Additionally, data quality was ensured through member checks, as participants were provided with the transcripts for their review and input within 7 days of the interview. Transcripts were exported to the NVivo V.12 software (QST International, Cambridge, MA, USA) and thematic analysis was performed (Dhakal, K. 2022)). Participants’ responses were coded into the parent, child, and sibling nodes for emerging themes and sub-themes. The node structure and coding matrix were reviewed by two investigators. A combined approach to data analysis inclusive of inductive and deductive reasoning was used to interpret emerging themes/sub-themes. Additionally, the NVivo tools were used to create a word cloud to visualize the technology type and utilization in LTC homes.

## Results

All participants were actively involved with the digital technology role out and discussed that they were responsible for decision-making pertinent to technology uptake and scale-up in their LTC homes. We found that the current use, needs/demands, and factors influencing the uptake of technologies varied by the size of the LTC home. We focus on three emerging themes and sub-themes below.

### Current Use and Needs/Demands of Digital Technologies

**Figure 2.**
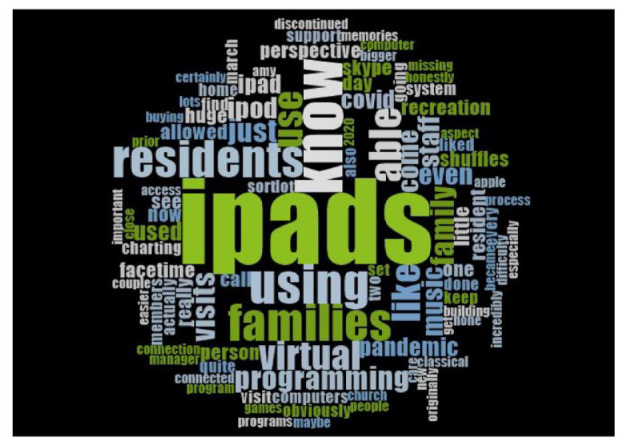
Word cloud displaying the frequency of words used by participants when discussing needs/demands for digital devices in long-term care homes. The size of the word indicates the frequency of use.

Almost all participants reported varying levels of digital technology use for the purposes of ongoing communication, recreational activities, and social engagement of residents in LTC homes. The most frequently used technologies included desk/laptop computers connected with Wi-Fi networks, television (TV), and radios. The use of iPads and cellular devices paired with data plans primarily depended on the available resources in a given LTC home (Figure 2). For example, a small-size home had a very limited number of iPads. Whereas large homes had already purchased some iPads pre-pandemic, and community members donated quite a few additional devices during the pandemic. Some participants revealed that iPads were also needed for virtual family visits, remote church services, and physician visits. A participant in a large LTC home mentioned:

Through the pandemic, residents demanded lots and lots of iPads to support virtual communication with family members. (Participant ID: 4)

Another participant added that “what we were finding with the iPads during our outbreaks and during COVID is that we also use it as a mechanism for programming so a person can leave the room, we could set them up with a church service or music or programming that they’d liked.” (Participant ID: 9)

### Facilitators/Enabling Factors Influencing Technology Uptake

Several participants highlighted regional-level, program-specific, and community-level factors that have had a significant impact on accessing and utilizing digital technologies in LTC homes during the COVID-19 pandemic.

#### Regional support system

The leadership and the information technology (IT) specialists provided ongoing system support with technology-associated procurement and installation. One participant reported:

We have a whole department behind us here to be able to support our IT (needs). (Participant ID:15)

Another confirmed the level of support commenting, “I think we’re fortunate as municipal homes, to have access to an IT department and so when we. . .want to have a look at how we integrate new technologies into what’s existing, there’s support for that.” (Participant ID: 4)

Leadership also provided necessary supplies for effective technology implementation. According to a participant, “we were extremely fortunate that our director, whatever the residents needed, there was very little no’s if we needed something. So, if we needed radios, or if we needed the TVs and the isolation rooms, or even if we would have said we needed iPads” (Participant ID: 9).

#### Staff redeployment

The Regional Municipality of Niagara redeployed staff from across the organization to the LTC homes during the pandemic to ensure that homes had sufficient resources to implement enhanced infection prevention and control measures and to help mitigate the impact of social isolation on residents. However, a number of redeployed staff supported virtual visits for LTC residents due to high volume of families who requested frequent virtual visits with residents. Study participants reported the benefits of redeployed staff, “it was kind of like a dream, compared to what we had before [in terms of a number of staff members].” (Participant ID: 1). The redeployed staff were key contributors to helping residents access technology to communicate with family members as often, “we were able to redeploy them to assist with things such as the virtual calls” (Participant ID: 12).

During COVID-19, LTC homes supported many family members to schedule virtual calls with residents on a daily/weekly basis. Participants in the large LTC homes reported having a lot of call volumes and they reported:

Because we’re such an extremely large home, it was very challenging to manage. And we have so many families and residents who have more than one contact. So, it became very, very challenging to be able to enable those calls. And because of the resources that we had, and the number of calls we had them scheduled in. . .we utilize redeployed staff as well as our recreation staff to facilitate those virtual calls. (Participant ID: 12)

Often, in LTC homes, older adults have trouble with using technology. However, redeployed staff acted as a facilitator for helping the older adults, as one participant said:

[For] most of our residents, the staff had to facilitate that call, they just couldn’t sort of give them a phone or an iPad and hook them up and leave the room. A lot of them had they did have to stay with us to facilitate that. So, it was very challenging to be able to manage those connections. I don’t have the numbers, but I know there were thousands and thousands of connections that were made. But we would not have been able to make those connections without the support of the redeployed staff that we had. (Participant ID: 12)

#### Community donations

Fundraising groups, family members of residents and local community organizations generously provided donations that helped LTC homes access extra digital technologies during the pandemic. With these donations, one participant reported “we were able to kind of draw on stuff [donations] if we needed anything” (Participant ID: 2); “they basically asked us to provide them a wish list of things that we would like, and technology is always on there, or iPads” (Participant ID: 12). Community donations helped to facilitate technology uptake in LTC by providing some technology that was needed during COVID-19, including the increased demand for iPads for virtual visits. All participants expressed gratitude toward larger community members for their generous donations, “we are really lucky; we’ve got a good volunteer base and a good donor base, and they’ll provide donations for technology that the residents use” (Participant ID: 4).

### Barriers/Impeding Factors Influencing Technology Uptake

Some participants reported issues related to the individual capacity to learn how to use new devices/programs and operational impediments with the use of technology in LTC homes during the COVID-19 pandemic.

#### The technology learning curve in older adults

A few participants mentioned issues related to residents’ limited knowledge and/or sub-optimal level of comfort independently using technologies “the challenges with our seniors. . . is the fact that they lacked the technology to be able to understand how to use those devices, number one, or are need large prints” (Participant ID: 12) and “in long term care, there are very few residents who can independently initiate, you know, a zoom call or FaceTime or what have you” (Participant ID: 4). Participants identified this as a challenge because, residents always needed staff members to assist them and with the influx of families scheduling virtual visits staff members had to spend a lot of time assisting residents facilitate these calls as well, there were residents who were not able to adapt to the technology: “We had very few who really didn’t take to it, we had a couple of residents who. . .because of their level of cognitive impairment, couldn’t process it (Participant ID: 4). This was challenging as during COVID-19 technology was the only way for residents to visit with family members due to government restrictions, leaving those who could not adapt to the technology with less family connection.

#### Technology-related bottlenecks

Almost all participants reported operational impediments, which were mainly due to the magnitude and intricacies of various technologies in LTC homes. Because of varying resources and the extent to which new technologies were introduced, most LTC homes went above and beyond, whereas some homes reported technical issues. One such big issue was with the Wi-Fi:

Sometimes the Wi-Fi signals are not very strong if it’s not on our regional system, versus our public system. So, the public system, say a family member came in and they wanted to show their loved one pictures on their iPad, their own personal iPad, or their cell phones, in our towns where they can log into our public Wi-Fi, and access that free of charge. But if they walk on into the residence room, the signal might drop, it might not be as strong. . . So that’s a challenge. (Participant ID: 1)

With the rapid pace of COVID-19 spread and higher mortality risks in the older population, LTC stakeholders needed to make tough choices in terms of resource allocation and budgeting for more devices. Although many participants reported the advantage of devices donated by the community members, there were some concerns with the make, model, type, and useful life of the devices donated. Some of these donated devices were fairly outdated and did not support audio/video capabilities. Additionally, there were IT challenges hooking these devices with a central secured network in the LTC home. One participant mentioned that “The biggest challenge is that because of how we’re set up for IT support within Niagara region and within the homes, is that all of our iPads and things need to come from our headquarters. . .So we did get donated iPads. But unfortunately, we’re not able to use them instantly.” (Participant ID: 12). Another participant added that “they can’t just take an iPad and put it on the network, obviously, because we have a lot of secure data here that’s at risk” (Participant ID: 14).

## Discussion

This study reports on the perspectives of digital technology use concerning managers and administrators in LTC homes and has demonstrated that factors related to access and utilization vary between small, mid, and large size homes in the Niagara Region. The large size LTC homes, however, face higher call volumes indicating the ever-increasing demand from the family/caregivers wanting to connect with their loved ones. Our findings further strengthen the policy advocacy favoring additional financial investments in technology solutions for virtual connection, communication, and social engagement among residents of LTC homes during the COVID-19 lockdowns. More importantly, the constant leadership support, redeployment of staff, and community donations are fundamental facilitators to promoting the technology uptake in LTC homes during the pandemic.

Technology-driven innovations specifically aiming to improve the care of the elderly in LTC homes present a timely opportunity to address the challenges associated with a changing demographic profile of Ontarians and the rising cost of care (Jaana & Paré, 2020). As our study highlighted, the use of digital technologies has been successful in minimizing many of the problems faced by elderly individuals during the pandemic and allowed many of these individuals to continue socializing, working, and accessing important aspects of their healthcare. However, the elderly population has also been disproportionately affected by some of the worst effects of the pandemic such as more stringent lockdown measures, and increased risks of mental and physical health problems, and the digital divide has seen that the effects of these measures have not been minimized (Martins Van Jaarsveld, 2020). It is projected that there will be nearly 238,000 Ontarians between the ages of 71 and 89 years by 2035 (Statistics Canada, 2010). Moreover, the gap between the number of LTC beds required and the number supplied will grow to between 57,000 and 127,000 by 2035 (Statistics Canada, 2010). Further, significant challenges related to human resources/staffing, financial and regulatory barriers, lack of sufficient funding, and the highly regulated nature of the LTC sector may limit the rate at which new technologies can be adopted (CBC, 2011). To meet the necessary demands and support for the LTC sector and the broader healthcare system, more research is warranted with respect to cost expenditures, allocations, and the effectiveness of technologies that support healthy aging (Jin et al., 2015). Previously conducted research documented the aggregated benefits of technology-driven interventions for improving the quality of life of older adults in home care (Akbar et al., 2018). Particularly, video calls are found to reduce loneliness and social isolation among residents of LTC homes (Zamir et al., 2018). Similarly, a systematic review highlighted mental health benefits associated with person-centered care assisted by technology use among older adults with depressive symptoms (Harerimana et al., 2019). There are notable differences in the methodology of how and where these previous studies were conducted. For example, most of these studies were conducted in either a single site or performed a secondary data analysis/review without addressing issues related to population heterogeneity. However, we would argue that a mixed methods approach targeting individual care homes (with a focus on care providers, residents, and their caregivers) will provide more compelling evidence of the economic and psychosocial impacts of technology uptake in small, mid, and large-size LTC homes.

In our study, participants continually emphasized the role of supportive leadership and community donations in leveraging the use of technologies for residents during the COVID-19 lockdowns in LTC homes. Several studies have documented the societal gains when local citizens are involved in the upliftment of the community (Gradinger et al., 2015). A common slogan, “nothing about us, without us,” (Stewart, 2017) may sound generic, but it fits well with the caring notion that the community offered to help residents in LTC homes during unprecedented times in the history of humankind. This is a unique finding of our study that is not well documented in the previous literature surrounding past pandemics. More research is needed to triangulate the incremental value of community donations to sustain technology infrastructure and overall health system gains in the near future.

We also found that residents’ cognitive impairment presents a challenge to implementing virtual visits during the pandemic. Our findings are corroborated by studies conducted elsewhere in which LTC homes with a limited number of iPads to share between residents, resulted in limited interaction with their family, friends, and volunteers (Chu et al., 2020). Many of the elderly residents also lacked the dexterity to hold a digital device such as an iPad, cellphone, or tablet steady and lacked the technical proficiency to use such devices (Chen et al., 2021). In most instances, this challenge could be addressed through one-to-one resident support provided by redeployed staff. While this study underscores the need for more recreational staff in LTC homes, there is compelling evidence of involving family/caregivers in clinical decision-making and social connection (Gaugler, 2005). A pre-pandemic Canadian study highlighted the significance of caregiver/family involvement in the planning and delivery of care for residents in LTC homes (Bauer & Nay, 2003). In that study, residents who had their family/caregivers involved in the trajectory of care demonstrated improvement in clinical conditions and positive mental health outcomes.

To our best knowledge, this is the first study to report on the administrative and managerial perspectives of technology use in LTC homes during COVID-19 at the regional level in Ontario and the rest of the Canadian provinces. Emerging findings contribute to the scientific knowledge regarding the current state of technology use, facilitators and barriers for further technology uptake as we move through COVID-19 waves. This study also raises important secondary research questions on evaluating the resident-level outcomes and experiences to inform decisions on resource allocation.

### Limitations and Conclusion

Our study used a qualitative exploratory study design, which has certain limitations for the time and place of data collection. First, this study was conducted approximately around wave 3 of the COVID-19 pandemic, so stakeholders have recall bias of the challenges associated with the early rollout of technologies. While this study added the perspective of managerial and administrative staff in publicly funded LTC homes across the region, there is a lack of transferability of findings to other LTC settings, which are exclusively privately operated, retirement homes, and home care in Canada. However, we believe these findings will bridge critical knowledge gaps surrounding technology uptake in LTC homes on the road to recovery from the COVID-19 pandemic. The importance of institutional leadership, financial commitment, and community support are integral to promoting technology uptake during the subsequent waves of COVID-19. While it is hard to predict the nature and extent of future restrictions for in-person family visits in LTC homes, there is a silver lining to existing technological infrastructure and effective utilization to promote mental health for residents in LTC homes. More research is needed to assess technology-related learning capabilities and remedial strategies for residents with cognitive or physical limitations.
